# Supplementary material for: Whole-Genome Analysis of Influenza A(H3N2) and B/Victoria Viruses Detected in Myanmar during the COVID-19 Pandemic in 2021
Source: Viruses. 2023 Feb 20;15(2):583. doi: 10.3390/v15020583 (PMC9964416; doi:10.3390/v15020583)
Supplement: Supplementary file 1 [file viruses-15-00583-s001.zip › Supplementary Tables_1-5.pdf]

Table S1. Strain names and accession numbers of eight segments of influenza A(H3N2) and B/Victoria viruses collected in Myanmar in 2021, sequenced during this study, and deposited in GISAID.

Strain name	ID	PB2	PB1	PA	HA	NP	NA	MP	NS
A/Myanmar/21M003/2021	EPI_ISL_13614323	EPI2082798	EPI2082799	EPI2082800	EPI2082801	EPI2082802	EPI2082803	EPI2082804	EPI2082805
A/Myanmar/21M004/2021	EPI_ISL_13614361	EPI2082806	EPI2082807	EPI2082808	EPI2082809	EPI2082810	EPI2082811	EPI2082812	EPI2082813
A/Myanmar/21M007/2021	EPI_ISL_13631518	EPI2082943	EPI2082944	EPI2082945	EPI2082946	EPI2082947	EPI2082948	EPI2082949	EPI2082950
A/Myanmar/21M008/2021	EPI_ISL_13631519	EPI2082951	EPI2082952	EPI2082953	EPI2082954	EPI2082955	EPI2082956	EPI2082957	EPI2082958
A/Myanmar/21M009/2021	EPI_ISL_13631520	EPI2082959	EPI2082960	failed	EPI2082961	EPI2082962	EPI2082963	EPI2082964	EPI2082965
A/Myanmar/21M013/2021	EPI_ISL_13631521	EPI2082966	EPI2082967	EPI2082968	EPI2082969	EPI2082970	EPI2082971	EPI2082972	EPI2082973
A/Myanmar/21M014/2021	EPI_ISL_13631522	EPI2082974	EPI2082975	EPI2082976	EPI2082977	EPI2082978	EPI2082979	EPI2082980	EPI2082981
A/Myanmar/21M017/2021	EPI_ISL_13631686	EPI2082982	EPI2082983	EPI2082984	EPI2082985	EPI2082986	EPI2082987	EPI2082988	EPI2082989
A/Myanmar/21M022/2021	EPI_ISL_13631687	EPI2082990	EPI2082991	EPI2082992	EPI2082993	EPI2082994	EPI2082995	EPI2082996	EPI2082997
A/Myanmar/21M073/2021	EPI_ISL_13631776	EPI2082998	EPI2082999	EPI2083000	EPI2083001	EPI2083002	EPI2083003	EPI2083004	EPI2083005
A/Myanmar/21M142/2021	EPI_ISL_13631777	EPI2083006	EPI2083007	EPI2083008	EPI2083009	EPI2083010	EPI2083011	EPI2083012	EPI2083013
A/Myanmar/21M143/2021	EPI_ISL_13631778	EPI2083014	EPI2083015	EPI2083016	EPI2083017	EPI2083018	EPI2083019	EPI2083020	EPI2083021
A/Myanmar/21M144/2021	EPI_ISL_13631779	EPI2083022	EPI2083023	EPI2083024	EPI2083025	EPI2083026	EPI2083027	EPI2083028	EPI2083029
B/Myanmar/21M015/2021	EPI_ISL_13631828	EPI2083031	EPI2083032	EPI2083030	EPI2083033	EPI2083034	EPI2083035	EPI2083036	EPI2083037
B/Myanmar/21M023/2021	EPI_ISL_13631829	EPI2083038	EPI2083039	EPI2083040	EPI2083041	EPI2083042	EPI2083043	EPI2083044	EPI2083045
B/Myanmar/21M024/2021	EPI_ISL_13632067	failed	failed	failed	EPI2083046	EPI2083047	EPI2083048	EPI2083049	EPI2083050

Abbreviations: PB2, polymerase basic 2; PB1, polymerase basic 1; PA, polymerase acid; HA, hemagglutinin; NP, nucleocapsid protein; NA, neuraminidase; MP, matrix protein; NS, nonstructural protein.

Table S2. Common amino acid substitutions in seven segments of A(H3N2) viruses collected in Myanmar 2021 and compared with those of the SH 2021 vaccine strains A/Hong Kong/45/2019.

		Amino acid substitutions																		
	**Segment**	**PB2**			**PB1**									**PA**			**NP**			
Vaccine strain	Clade	112	415	449	314	400	561	571	587	603	618	619	692	321	402	626	136	220	418	500
A/Hong Kong/45/2019	3C.2a.1b.1b	N	M	I	S	A	D	R	G	G	M	R	S	Y	S	R	I	E	L	G
Myanmar viruses 2021	2a.3	D	V	V	N	E	G	Q	R	E	T	I	N	C	T	K	M	D	I	E
	**Segment**	**NA**								**MP**				**NS**						
Vaccine strain	Clade	45	54	315	329	344	346	463	465	271	285	298	299	135	211	218	241			
A/Hong Kong/45/2019	3C.2a.1b.1b	L	E	S	S	E	G	D	N	V	P	S	T	R	N	P	N			
Myanmar viruses 2021	2a.3	P	G	R	N	K	D	N	S	M	L	C	A	K	H	L	D			

Abbreviations: SH, Southern Hemisphere; PB2, polymerase basic 2; PB1, polymerase basic 1; PA, polymerase acid; NP, nucleoprotein; NA, neuraminidase; MP, matrix protein; NS, nonstructural protein.

Table S3. Common amino acid substitutions in seven segments of A(H3N2) viruses collected in Myanmar 2021 and compared with those of the SH 2022 vaccine strains A/Darwin/6/2021.

		**Amino acid substitutions**												
	**Segment**	**PB1**			**PA**					**NA**		**MP**		
Vaccine strain	Clade	376	583	619	99	321	402	407	660	54	329	288	298	325
A/Darwin/6/2021	2a	T	R	T	E	Y	A	V	S	E	S	H	T	V
Myanmar viruses 2021	2a.3	A	K	I	G	C	T	I	A	G	N	P	A	I

Abbreviations: SH, Southern Hemisphere; PB2, polymerase basic 2; PB1, polymerase basic 1; PA, polymerase acid; NP, nucleoprotein; NA, neuraminidase; MP, matrix protein; NS, nonstructural protein.

Table S4. Common amino acid substitutions in seven segments of B/Victoria viruses collected in Myanmar 2021 and compared with those of the SH 2021 vaccine strains B/Washington/02/2019.

		**Amino acid substitutions**																		
	**Segment**	**PB2**	**PB1**		**PA**				**NP**			**NA**								
Vaccine strain	Clade	56	375	454	272	312	613	709	549	550	551	42	71	344	345	396	402	465	470	484
B/Washington/02/2019	V1A.3	S	S	N	H	K	K	F	R	D	T	P	V	Q	R	N	G	H	E	V
Myanmar viruses 2021	V1A.3a.2	N	G	D	Y	R	R	V	G	S	R	Q	A	R	G	S	D	Y	G	I
	**Segment**	**MP**					**NS**													
Vaccine strain	Clade	15	285	306	362	378	130	306	311	312										
B/Washington/02/2019	V1A.3	T	I	D	F	V	L	N	L	H										
Myanmar viruses 2021	V1A.3a.2	I	T	G	S	A	F	S	P	N										

Abbreviations: SH, Southern Hemisphere; PB2, polymerase basic 2; PB1, polymerase basic 1; PA, polymerase acid; NP, nucleoprotein; NA, neuraminidase; MP, matrix protein; NS, nonstructural protein.

Table S5. Common amino acid substitutions in seven segments of B/Victoria viruses collected in Myanmar 2021 and compared with those of the SH 2022 vaccine strains B/Austria/1359417/2021.

		**Amino acid substitutions**											
	**Segment**	**PB2**	**PB1**		**PA**			**NP**			**NA**		**NS**
Vaccine strain	Clade	396	51	375	272	376	613	28	550	551	455	460	312
B/Austria/1359417/2021	V1A.3a.2	I	D	S	H	I	K	A	D	T	V	Y	H
Myanmar viruses 2021	V1A.3a.2	M	N	G	Y	M	R	T	S	R	A	C	N

Abbreviations: SH, Southern Hemisphere; PB2, polymerase basic 2; PB1, polymerase basic 1; PA, polymerase acid; NP, nucleoprotein; NA, neuraminidase; MP, matrix protein; NS, nonstructural protein.
