# Supplementary figures and images for: Whole-Genome Analysis of Influenza A(H3N2) and B/Victoria Viruses Detected in Myanmar during the COVID-19 Pandemic in 2021
Source: Viruses. 2023 Feb 20;15(2):583. doi: 10.3390/v15020583 (PMC9964416; doi:10.3390/v15020583)

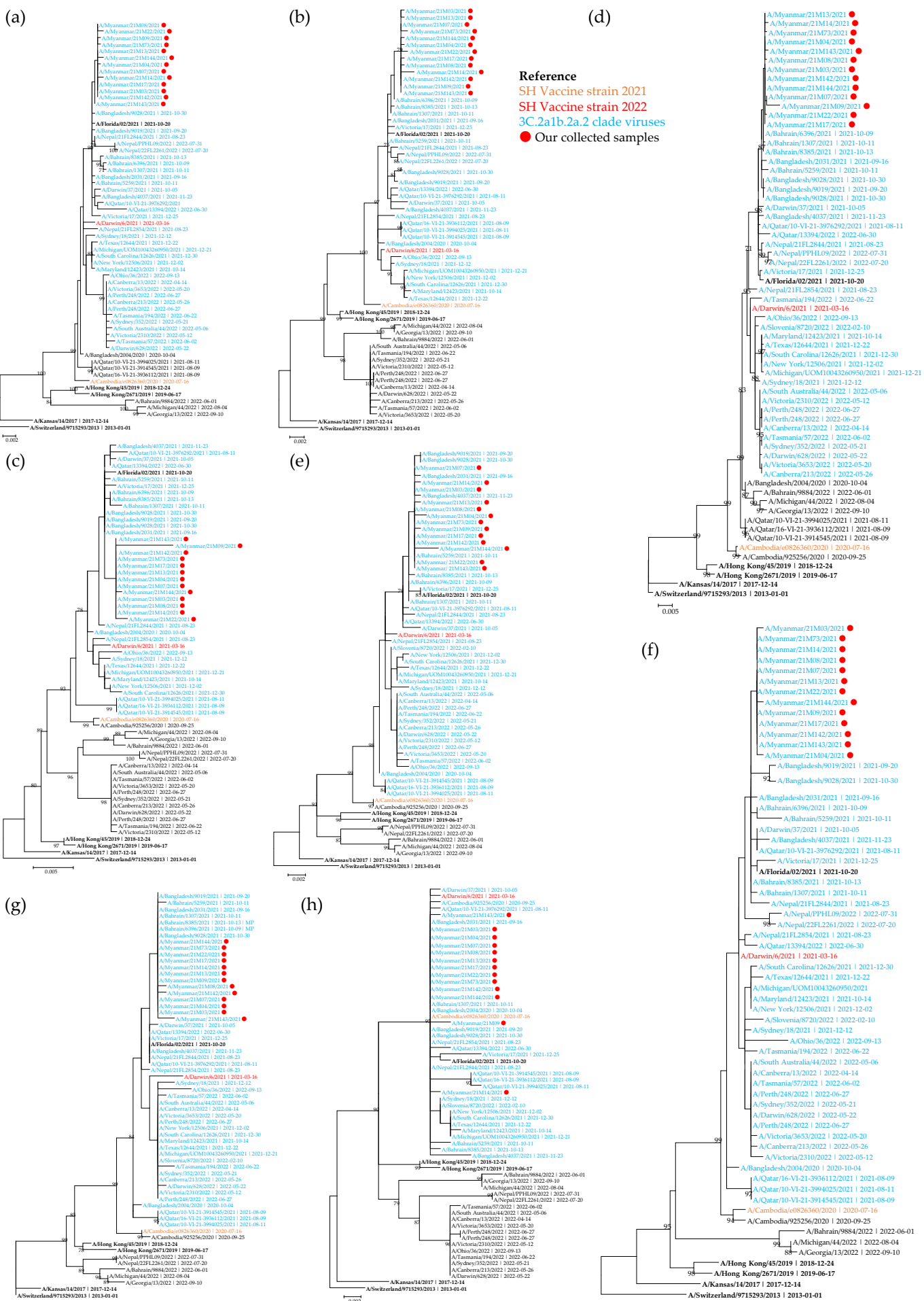

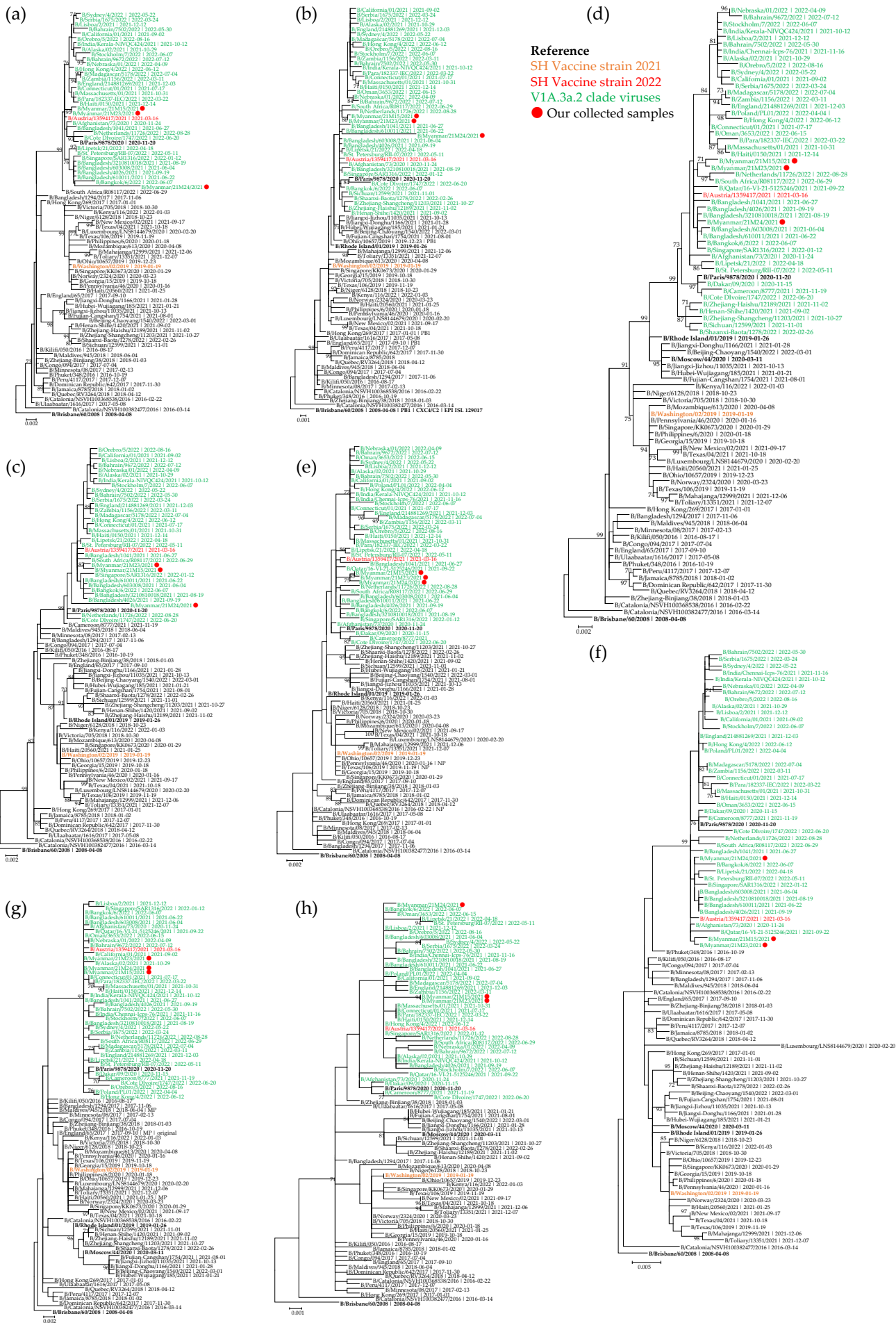

Supplement: Supplementary file 1 [file viruses-15-00583-s001.zip › Supplementary Figures_1-2.pdf]
